# PD-L1 and MRN synergy in platinum-based chemoresistance of head and neck squamous cell carcinoma

**DOI:** 10.1038/s41416-019-0697-x

**Published:** 2019-12-19

**Authors:** Bin Shen, Dongyan Huang, Andrew J. Ramsey, Kevin Ig-Izevbekhai, Kevin Zhang, Shayanne A. Lajud, Bert W. O’Malley, Daqing Li

**Affiliations:** 1 Department of Otolaryngology–Head and Neck Surgery, University of Pennsylvania School of Medicine, Philadelphia, PA 19104 USA; 2 Department of Otolaryngology-Head and Neck Surgery, Shanghai General Hospital, Shanghai, China; 3 Department of Otolaryngology-Head and Neck Surgery, PLA General Hospital, Beijing, China; 4 Department of Otolaryngology-Head and Neck Surgery, University of Puerto Rico School of Medicine, San Juan, Puerto Rico

**Keywords:** Head and neck cancer, Cancer therapeutic resistance, Targeted therapies, Double-strand DNA breaks, DNA damage response

## Abstract

**Background:**

We have been investigating the molecular mechanisms of cisplatin-induced chemoresistance in head and neck squamous cell carcinoma (HNSCC). Based on our previous findings, the present study investigates how the Mre11, Rad50, and NBS1 (MRN) DNA repair complex interacts at the molecular level with the programmed cell death ligand 1 (PD-L1) in cisplatin-induced chemoresistance.

**Methods:**

Human HNSCC cell lines were used to determine the role played by PD-L1 in cisplatin resistance. Initial experiments investigated PD-L1 expression levels in cells exposed to cisplatin and whether PD-L1 interacts directly with the MRN complex. Finally, in vitro studies and in vivo experiments on BALB/c nu/nu mice were performed to determine whether interference of PD-L1 or NBS1 synthesis modulated cisplatin resistance.

**Results:**

Exposure to cisplatin resulted in PD-L1 being upregulated in the chemoresistant but not the chemosensitive cell line. Subsequent co-immunoprecipitation studies demonstrated that PD-L1 associates with NBS1. In addition, we found that the knockdown of either PD-L1 or NBS1 re-sensitised the chemoresistant cell line to cisplatin. Finally, but perhaps most importantly, synergy was observed when both PD-L1 and NBS1 were knocked down making the formerly chemoresistant strain highly cisplatin sensitive.

**Conclusions:**

PD-L1 plays a pivotal role in cisplatin resistance in chemoresistant human HNSCC cell lines.

## Background

Head and neck cancer (HNC) is the sixth most common cancer worldwide, accounting for >350,000 deaths annually,^1,2^ with >65,000 HNC diagnoses expected within the United States in 2019.^3^ HNCs are mostly squamous cell neoplasms that originate from the epithelial lining of the upper aerodigestive tract and are commonly referred to as head and neck squamous cell carcinoma (HNSCC). While HNSCC is curable when diagnosed early, the prognosis is very poor when diagnosed at an advanced stage.^4^ The 3-year disease-free survival rate ranges between 35% and 55% across all stages, and there has not been a significant survival improvement over the past 30 years due to limited available approaches.^5^ Therefore, it is critical to understand the cause of treatment failure and to identify molecular mechanisms that can assist in the design of better and more effective therapeutic approaches to improve patients’ outcomes.

*Cis*-diamminedichloroplatinum(II) (cisplatin) is a platinum-based chemotherapy agent commonly used in combination with other drugs in the treatment of several types of human cancers, including HNSCC. Cisplatin induces apoptosis by multiple mechanisms including the induction of DNA damage, which overwhelms the cancer cell’s DNA repair mechanisms. However, repeated treatment cycles often lead to acquired platinum-based chemoresistance of cancer cells. This results in the use of higher doses of the drug, which can cause severe toxicities.^6–8^

The Mre11, Rad50, and NBS1 (MRN) complex plays an essential role in the cellular response to double-stranded DNA breaks.^9^ The complex identifies and binds to both ends of a double-stranded break and recruits other proteins associated with either the non-homologous end joining or homologous repair pathways. Increased MRN activity enhances the cells’ ability to repair DNA damage caused by various chemotherapies, including cisplatin treatment, and has been detected in a range of cancerous cells.^10^ Importantly, overexpression of the MRN complex proteins is associated with cisplatin resistance.^11,12^ In line with these findings, we have previously demonstrated that the disruption of the MRN complex sensitises HNSCC to cisplatin in vitro and in vivo through the dual disruption of DNA repair and telomere maintenance mechanisms.^11,13–16^

The membrane-bound protein programmed cell death receptor 1 (PD-1) has been implicated in a second resistance mechanism. The protein is located primarily on the immune system’s T cells. When the protein binds its ligand, PD-L1, T cells are inactivated either through anergy or by undergoing apoptosis resulting in the PD-L1-bound cell being immunologically privileged.^17^ Abnormal levels of PD-L1 expression have been found in many cancers, including HNSCC, which may result in unhindered tumour growth.^18–20^ Recent studies suggest an alternative function of PD-L1. PD-L1 has been observed translocating from the cell surface to the nucleus of breast cancer cells following doxorubicin therapy.^21^ Preliminary studies from our laboratory have revealed the overexpression of PD-L1 and its presence in the nucleus of chemoresistant JHU006 human HNSCC cells following cisplatin treatment. While a synergy has been identified between cisplatin and PD-1/PD-L1 inhibition in HNSCC,^22^ its mechanism remains largely unknown.

In this study, we hypothesised that a link exists between the translocated PD-L1 and the proteins of the MRN complex in the development of a cisplatin-resistant phenotype. We used the chemoresistant JHU006 and chemosensitive JHU020 cell lines, which have been genetically characterised and whose MRN expression levels are known, in order to test whether PD-L1 binds to the MRN complex and whether synergies exist between the two mechanisms of chemoresistance. We also investigated whether small interfering RNA (siRNA)-based knockdown of PD-L1 and MRN could reverse cisplatin chemoresistance. Finally, we performed in vivo experiments on mice in order to determine the effects of these knockdowns on HNSCC tumour size. The ability to re-sensitise cancer cells in a clinical setting could have a major impact on existing chemotherapeutic regimens.

## Methods

### Cell culture

JHU020 and JHU006 human HNSCC cell lines were generated and genetically characterised at the Johns Hopkins University Head and Neck Biological Research Laboratories from human tumour explants. Both cell lines were propagated and carefully maintained in our laboratory. A large batch of both JHU020 and JHU006 cell lines were produced immediately prior to the beginning of the study. The majority of the cells were frozen while aliquots were sent for authentication using Short Tandem Repeat Analysis by BioSynthesis Inc. The cells were also tested for mycoplasma contamination. With the exception of those experiments that took place simultaneously, an aliquot of each cell line was thawed for every experiment. Long-term propagation did not occur. The cell lines were cultured in RPMI-1640 culture media (Gibco, Baltimore, MD), supplemented with 12% Foetal Bovine Serum (Hyclone Laboratories, South Logan, UT) and 1% Penicillin–Streptomycin solution (Corning, Manassas, VA). The cells were plated in 6-well plates at a density of approximately1.5 × 10^5^ cells/ml for JHU006 cells and 3 × 10^5^ cells/ml for JHU020 cells.

### Cisplatin concentration

Unless otherwise stated, the in vitro cisplatin (Teva Pharmaceuticals, Sellersville, PA) experiments using the JHU020 and JHU006 cells lines were performed at the cells’ half maximal inhibitory concentrations (IC50) of 0.3 and1.6 µg/ml, respectively.

### Animals

Animal experiments were performed on 6-week-old, drug-naive BALB/c nu/nu mice (The National Cancer Institute). Mice were housed in filter-top cages in a designated animal facility, with four same-sex mice per cage. In accordance with the University of Pennsylvania Institutional Animal Care and Use Committee statutes, mice were given food and water ad libitum and monitored to ensure they maintained weight within 20% of age-matched controls. At the conclusion of experimental analysis, animals were euthanised by cervical dislocation.

### Co-immunoprecipitation (Co-IP)

Co-IP was performed according to the manufacturer’s instructions (Abcam Cambridge, MA, USA). Cell lysis buffer was applied to cells before the cells were transferred to a microcentrifuge tube and incubated with mixing for 30 min at 4 °C to allow the cells to lyse. The samples were then centrifuged to remove cell debris. Lysates from JHU006 and JHU020 cell lines were incubated with monoclonal mouse anti-human PD-L1 (Santa Cruz Biotechnology, Dallas, TX) at a concentration of 1–2 μg per 100–500 μg of total protein for 3–4 h at 4 °C. The PD-L1 was then captured using Protein A/G Sepharose beads. The beads were then thoroughly washed to remove the unbound protein. After elution from the beads, the immunoprecipitates were analysed by western blot.

### Treatment for siRNA and adenovirus short hairpin RNA (shRNA) experiment

The shRNA-PD-L1 and shRNA-NBS1 viruses were constructed according to our previously described methodology.^23^ Both JHU006 and JHU020 cell lines were treated with siRNA-STAT1 and siRNA-STAT3 at concentrations of 10 nM and siRNA-NBS1 and siRNA-PD-L1 at concentrations of 25 nM. Cells treated with control siRNA were used as experimental controls. The siRNA was combined with HiPerFect Transfection Reagent (QIAGEN, Germantown, MD) and was allowed to incubate for 20 min to allow the transfection complexes to form. Opti-MEM Reduced Serum Media (Gibco, Baltimore, MD) was then added to the RNA complexes, and 500 μl of the RNA complex-containing media was added to each well. After 4 h, another 1 ml of Opti-MEM was added to the remaining wells. The cells were collected at time points of 0, 12, 24, 48, and 72 h in preparation for western blot analysis designed to determine the optimal incubation time. The cells treated with the control siRNA treatment cells were collected at the 72-h time point.

### Alamar Blue cytotoxicity assay

JHU020 and JHU006 cells were loaded onto wells of 96-well plates at a density of 2 × 10^5^ cells/ml and 1 × 10^5^ cells/ml, respectively. The treatment groups consisted of 25 nM of siRNA-Control, 25 nM of siRNA-NBS1, 25 nM of siRNA-PD-L1, or 25 nM of a mixture of siRNA-NBS1 and siRNA-PD-L1 and a control using standard growth medium. HiPerFect Transfection Reagent at 0.75 μl/well was added to all wells containing siRNA. After 24-h incubation, cisplatin was added to half of the wells to give a final concentration equal to the cells’ IC50 concentration. Meanwhile, 0.75 μl/well of regular media was added to the remaining wells. Each experimental condition was run in quadruplicate on each plate. After incubation for a further 48 h, 10 μl of Alamar Blue cytotoxicity assay solution (Bio-Rad, Hercules, CA) was added to each well. The wells were incubated for a total of 4 h before the sample’s absorption at 570 and 630 nm was measured.

### Xenograft tumours in vivo

BALB/c nude mice were allocated to five treatment groups and one control group by non-blinded randomisation, with six mice in each group. In all groups, mice underwent surgical implantation and harvesting of HNSCC tissue. All surgical procedures were performed with animals under anaesthesia, using aseptic surgical techniques in a special procedure room. For all surgeries, a ketamine/xylazine cocktail was prepared at a concentration of 10/1 mg/ml and 0.010 ml/g was injected into the peritoneal cavity with a small needle, to achieve appropriate anaesthesia. Mice were monitored during recovery from anaesthesia and every 24 h after surgery. If mice demonstrated signs of pain upon recovery, they were treated with buprenorphine 0.05 mg/kg.

Tumours were established in the right flank of mice by subcutaneous injection of 1.0 × 10^7^ JHU006 cells. Seven days after tumour injection, mice underwent surgical exposure of neoplastic tissue. A pre-operative subcutaneous injection of 0.01 ml/g of 0.2 mg/ml bupivacaine was given at the site of the incision, then 0.01 ml/g of 0.005 mg/ml buprenorphine was administered. Finally, 0.01 ml/g of 0.5 mg/ml meloxicam was given subcutaneously before ketamine/xylazine anaesthesia. The tumours were then surgically exposed and measured in three dimensions. Subsequently, intratumoural injections containing 3.4 × 10^10^ plaque-forming units of adenoviruses encoding dominant-negative mutants of PD-L1 and NBS1 were delivered in 50 µl of saline, while 50 µl of saline alone were delivered to the control tumours. Cisplatin was intraperitoneally injected into the cisplatin-treated groups at 5 mg/Kg. Eight days later, the tumour masses were measured again in three dimensions and harvested using the aforementioned anaesthetic dosing, with individual mice as the units of analysis. All procedures were daytime experiments. All animal experiments were performed in accordance with protocols approved by the Animal Care and Use Committee of The University of Pennsylvania.

### Statistical analysis

Mann–Whitney analysis was applied using STATMOST (Detaxion Software Inc., Los Angeles, CA, USA) to determine the statistical significance.

## Results

### Cisplatin upregulates the expression of PD-L1 at the protein and mRNA levels in JHU006 cells, but not in JHU020 cells

**Fig. 1:**
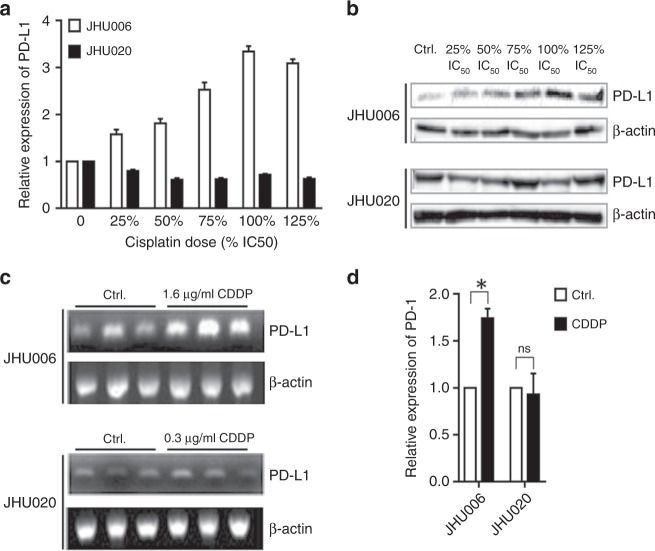
PD-L1 expression increased in chemoresistant cells following exposure to cisplatin. JHU020 and JHU006 cells were incubated with a range of cisplatin concentrations for 48 h (**a**, **b**). The addition of cisplatin resulted in a significant upregulation of PD-L1 protein expression in the chemoresistant JHU006 cells with a near-linear relationship observed between PD-L1 expression and cisplatin concentration at cisplatin levels at or below the IC50. However, the level of PD-L1 expression did not change significantly upon cisplatin addition to the chemosensitive JHU020 cell line. **c**, **d** RNA was extracted from cells incubated in media with or without CDDP for 48 h. The quantification of the western blot bands indicates that application of cisplatin results in increased PD-L1 mRNA transcription in JHU006 cells but not in JHU020 cells (*P < 0.05). The statistics represent the standard errors of the means from three separate experiments. The western blots are cropped in order to improve clarity.

JHU006 and JHU020 cells were exposed to a range of cisplatin concentrations based on their respective IC50. Figure 1a, b demonstrate that the exposure of the chemoresistant JHU006 cells to cisplatin resulted in greatly increased PD-L1 expression, which occurred in a nearly dose-dependent manner. The highest level of PD-L1 expression was observed at the IC50 concentration where PD-L1 expression was almost threefold higher than the expression observed in the absence of cisplatin. In contrast, PD-L1 expression in the chemosensitive JHU020 cells was not significantly altered following their exposure to the drug. The effect of cisplatin on PD-L1 mRNA levels was also measured. Figure 1c, d demonstrate that the application of cisplatin to JHU006 cells resulted in almost a doubling of PD-L1 mRNA synthesis while the application of the drug to JHU020 cells had no significant effect on their PD-L1 mRNA levels. The differential expression of PD-L1 at the mRNA and protein levels in the chemosensitive and chemoresistant cell lines strongly implicate PD-L1 in the development of cisplatin resistance.

### PD-L1 associates with NBS1

**Fig. 2:**
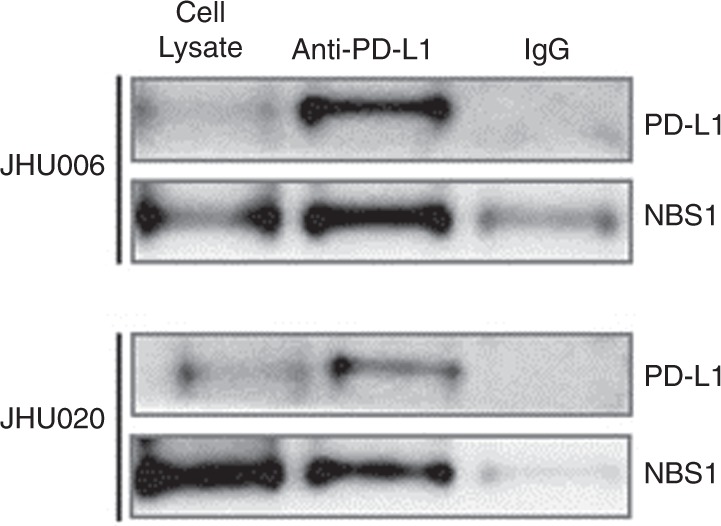
Co-immunoprecipitation of PD-L1 and NBS1. The western blot image (*n* = 3) indicates that anti-PD-L1 pulled down NBS1 from cell lysates obtained from both JHU020 and JHU006 cell lines. This indicates that PD-L1 is associated with either NBS1 or the MRN complex. The figure also demonstrates that much greater amounts of both NBS1 and PD-L1 were pulled down by the PD-L1 antibody from the chemoresistant JHU006 cell lysates than with the chemosensitive JHU020 lysates. The western blots are cropped in order to improve clarity.

Co-IP was then used to monitor the interactions between PD-L1 and the MRN complex. Figure 2 indicates that the PD-L1 antibody successfully pulled down NBS1 from both JHU006 and JHU020 cell lysates demonstrating that PD-L1 associates with NBS1 in both chemosensitive and chemoresistant cell lines. This strongly suggests that PD-L1 and NBS1 may regulate a common pathway.

### PD-L1 knockdown induced the reduction of phosphorylation of Akt and epidermal growth factor receptor (EGFR) expression in chemoresistant JHU006 cell lines

**Fig. 3:**
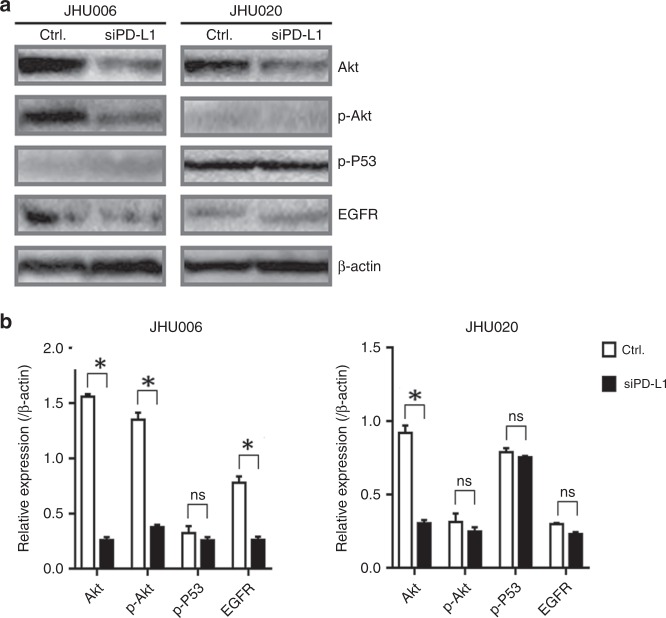
siPD-L1 induced a reduction of p-Akt and EGFR expression in the JHU006 chemoresistant cell line but not in chemosensitive JHU020 cells. **a** The expression of Akt, p-Akt, p-P53 and EGFR 48 h after the application of siPD-L1. **b** The intensities of the protein bands illustrated in **a** were quantified using the ImageJ program. **b** demonstrates that the addition of siRNA-PD-L1 to the cells resulted in statistically significant decreases in Akt, p-Akt and EGFR expression in the JHU006 cell line (*P < 0.05). Akt was the only one of the four proteins examined to show a significant reduction in expression in the JHU020 cell line (*P < 0.05). The statistics represent the standard errors of the means from three separate experiments. The western blots are cropped in order to improve clarity.

JHU006 and JHU020 cells were treated with siRNA-PD-L1 for 48 h before protein extraction and western blotting. The levels of Akt, p-Akt, p-P53 and EGFR expression were then determined. Figure 3a demonstrates that PD-L1 knockdown resulted in the qualitative downregulation of Akt, p-Akt and EGFR in JHU006 cells. Meanwhile, only Akt was significantly downregulated by PD-L1 in JHU020 cells. Quantitative data (Fig. 3b) confirmed that the downregulation of Akt, p-Akt and EGFR expression in the JHU006 cells lines was statistically significant. Conversely, only Akt expression was downregulated in the JHU020 cell line. Notably, the addition of siRNA-PD-L1 did not modify the expression levels of the anti-apoptotic protein p-P53 in either the JHU006 or the JHU020 cell lines.

### Signal transducer and activator of transcription 1 (STAT1) and STAT3 regulate PD-L1 expression independent of NBS1 in HNSCC cell lines

**Fig. 4:**
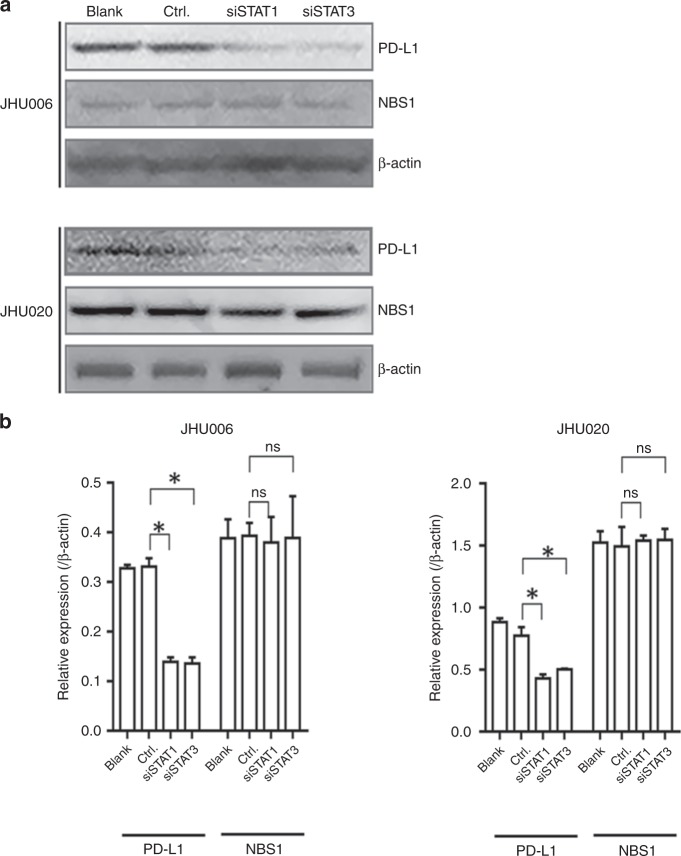
PD-L1, but not NBS1, expression correlates with the knockdown of STAT1 or STAT3 expression in both chemoresistant and chemosensitive cell lines. PD-L1 but not NBS1 was qualitatively downregulated in both JHU006 and JHU020 cell lines 48 h after siRNA-mediated knockdown of STAT1 or STAT3. The quantitative data demonstrate that there were statistically significant differences in PD-L1 expression in both cell lines between either the siRNA-STAT1- or siRNA-STAT3-treated samples and the control sample (*P < 0.05). The expression of NBS1 was unchanged in both cell lines following the application of siRNA-STAT1 or siRNA-STAT3. The statistics represent the standard errors of the means from three separate experiments. The western blots are cropped in order to improve clarity.

The STAT family has been implicated in the regulation of PD-L1.^24^ Consequently, we investigated the effect of downregulating STAT1 and STAT3 in both JHU006 and JHU020 cell lines. The expression of PD-L1 and NBS1 was monitored 48 h after siRNA-STAT1 or siRNA-STAT3 treatment. Figure 4a, b demonstrate that downregulation of STAT1 and STAT3 via siRNA, in turn, significantly downregulated PD-L1 but not NBS1 in both cell lines. The data suggest that both STATs are implicated in the regulation of PD-L1 expression independent of NBS1.

### Downregulation of PD-L1 and NBS1 re-sensitises chemoresistant cells to cisplatin therapy in vitro

**Fig. 5:**
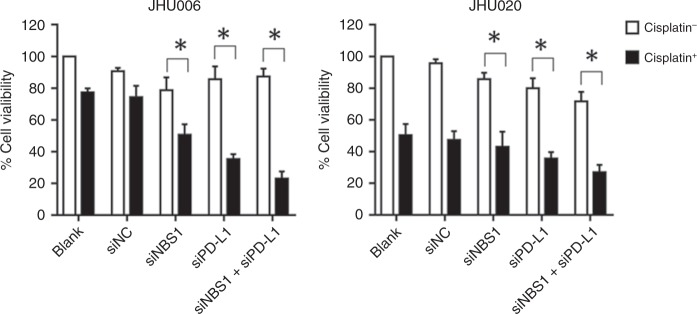
The effect of siRNA and cisplatin on cell viability. Cells that were not treated with cisplatin, regardless of cell type, maintained high levels of cell viability. All three cisplatin-treated JHU006 test groups displayed significant decreases in cell viability, compared to two cisplatin-treated control groups. The application of the mixture of the two siRNAs followed by exposure to cisplatin proved to be the most effective regimen, resulting in 80% cell death. While the addition of cisplatin to the JHU020 cell line resulted in a large decrease in cell viability, the addition of the siRNAs produced only a small further decrease in viability (*P < 0.05). The statistics represent the standard errors of the means from three separate experiments (SiNC control siRNA).

JHU006 and JHU020 cells were transfected with siRNA sequences that knocked down PD-L1 or NBS1 expression or treated with a siRNA mixture that knocked down both proteins. Cells were then exposed to their IC50 concentration of cisplatin and incubated for 48 h. The control group did not receive cisplatin. The cells’ viability was then determined using the Alamar Blue cytotoxicity assay. As shown in Fig. 5, siRNA treatments had a minimal effect on the viability of either JHU006 or JHU020 cells in the absence of cisplatin. When exposed to cisplatin and siNBS1, JHU006 cells had a 50% reduction in their viability. Interestingly, the application of siPD-L1 and cisplatin combination treatment of JHU006 cells appeared to be more effective, causing approximately a 65% reduction in cell viability (*P* < 0.05). It was highly significant that the use of a mixture of both siRNAs in combination with cisplatin proved to be the most effective treatment, resulting in approximately 80% cell death. This demonstrates synergy between the PD-L1 and NBS1/MRN. Conversely, the addition of cisplatin to siRNA-treated JHU020 cells produced much smaller decreases in cell viability as compared with JHU006 cells.

### Adenovirus-delivered shRNA for PD-L1 and NBS1 re-sensitise JHU006 cells to cisplatin therapy in vivo

**Fig. 6:**
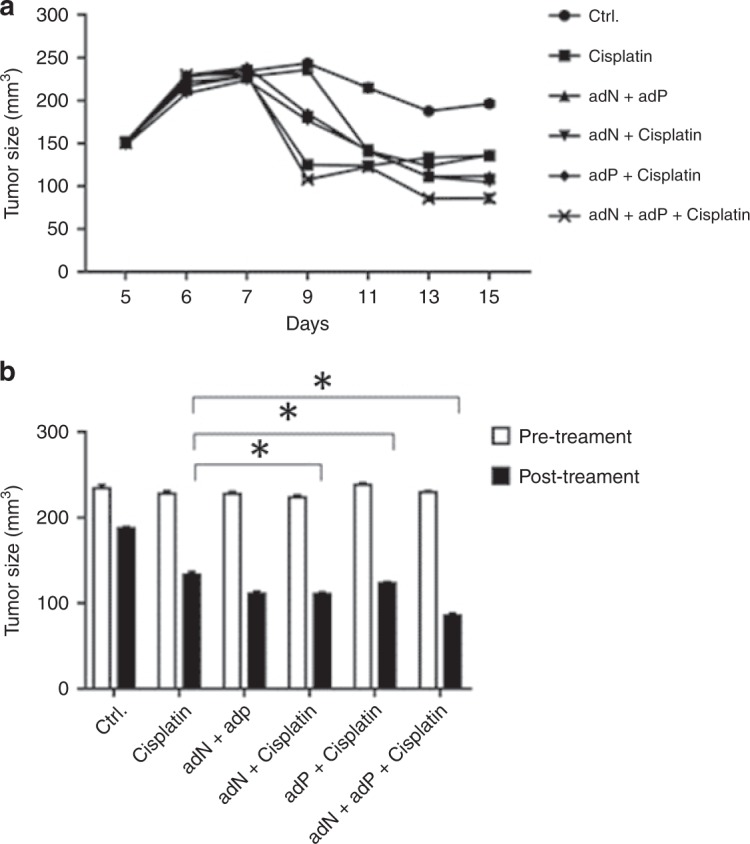
PD-L1 and NBS1 knockdown sensitises JHU006 HNSCC cells in vivo. **a** The change in the tumour volume following a range of treatments was followed over time. **b** The tumour volumes were measured pre-treatment (white) and post-treatment (black), at 15 days after tumour establishment. The addition of cisplatin alone resulted in a large decrease in tumour size. This decrease was enhanced by the application of adenoviruses. It was noticeable that the mice that received the adenoviral mixture experienced the largest decrease in tumour size (*P < 0.05). The statistics represent the standard errors of the means from three separate experiments.

All mice were drug-naive and healthy prior to surgery. The rodents were split into six treatment groups and then implanted with JHU006 tumour xenografts that were allowed to grow for 1 week before treatment was implemented. Tumours were harvested from six mice in each group. As shown in Fig. 6a, b, tumour volumes decreased for all groups, including the control, after treatment. The experimental groups that were treated with cisplatin and the mixture of shRNA adenoviruses that inhibited both PD-L1 and NBS1 expression showed the greatest reduction in volume (*P* < 0.05), again suggesting synergy between the two proteins.

## Discussion

While the exposure of chemoresistant JHU006 cells to cisplatin resulted in increased expression of PD-L1 both at the protein and mRNA levels, this pattern is not observed in the chemosensitive JHU020 cells, where cisplatin exposure did not significantly alter PD-L1 expression. These two patterns of expression strongly implicate PD-L1 in cisplatin chemoresistance in HNSCC. These results suggest that PD-L1 overexpression, which contributes to the immunosuppression of HNSCC, could be a promising therapeutic target. This finding is consistent with the literature where Cacan^25^ found that the expression of the immunosuppressive molecule PD-L1 is higher in chemoresistant cells than the parental chemosensitive ovarian cancer cells.

Given the known importance of chemoresistance in the prognosis of HNC, many studies have sought to discover ways to reverse the chemoresistant phenotype. Our previous studies^13–16^ identified multiple therapeutic targets involved in DNA repair pathways, including the sensitisation of cancer cells to chemotherapy and radiotherapy through the molecular disruption of the MRN complex, as well as through poly (ADP-ribose) polymerase inhibition.^26^ The NBS1 protein ensures the overall stability of the MRN complex and the knockdown of NBS1 can produce decreased expression of the other two members of the complex. In line with these crucial roles, we have previously demonstrated a significant synthetic lethality effect upon the dual disruption of NBS1 and (ADP-ribose) polymerase 1 in a novel clinically applicable approach. In the present study, the Co-IP of NBS1 by anti-PD-L1 indicates that PD-L1 associates with either NBS1 or the MRN complex and may play a major role in the development of chemoresistance. Similar to the findings of Sato et al.,^27^ we demonstrate an association between PD-L1 with MRN complex, suggesting a novel interaction between DNA repair and the mechanisms of immune response. Further research should be focused on this area to test the exact mechanism of this interaction.

The STAT signalling pathway is known to play an important role in many cellular processes, including division, apoptosis and metastasis.^28^ The transcriptional activator STAT3 has been reported to be involved in the development and growth of tumours,^29^ while PD-L1 is one of the proteins regulated by STAT3 in tolerogenic antigen-presenting cells.^30^ STAT3 inhibition has also been recently shown to reduce PD-L1 expression in non-small cell lung cancer.^31^ Similarly, our present experiments demonstrate that the inhibition of STAT3 expression resulted in decreased PD-L1 expression in both cell lines. Unexpectedly, inhibiting STAT1 expression also decreased the PD-L1 expression in both cell lines. Our data also indicate that both the Akt/STAT3 and the JAK/STAT1 pathways regulate PD-L1 expression. Elucidating the role played by STAT1 and STAT3 in the regulation of PD-L1 in HNSCC may shed light on potential therapeutic targets or factors influencing the ability of these tumours to respond to a PD-L1 blockade. Although the knockdown of either STAT1 or STAT3 reduces PD-L1 expression, it does not affect NBS1 expression. This suggests that the regulation of PD-L1 and NBS1 probably occurs via independent mechanisms.

Interestingly, the JHU006 and JHU020 cell lines showed different protein expression profiles following the knockdown of PD-L1. The knockdown of PD-L1 resulted in lower expression of both P-Akt and EGFR in JHU006 cell lines, but not in JHU020 cells. Thus the data strongly suggest that both Akt and EGFR are downstream of PD-L1. The siRNA-mediated knockdown of PD-L1, NBS1 or both proteins followed by exposure to cisplatin significantly decreased cell viability. The cell viability assays also demonstrated that targeting the expression of PD-L1 and NBS1 can re-sensitise JHU006 cells to cisplatin therapy. The fact that the experimental groups produced different levels of re-sensitisation suggests that multiple pathways could be involved. Remarkably, both the cell viability assays and in vivo studies indicate that downregulation of both PD-L1 and NBS1 in combination with cisplatin leads to the most potent antitumour effect. This indicates the presence of a synergy between the two proteins and suggests that they may collectively contribute to the chemoresistant phenotype.

Membrane PD-L1 expression in tumours is already known to result in a poor prognosis. This is due to the creation of an immunologically privileged site caused by the interactions of PD-L1 with PD-1 receptors on T cells. PD-L1 has the potential to be a major dual therapeutic target. For instance, if cisplatin induces PD-L1 upregulation, PD-L1 could become an immunotherapy target, stimulating the immune system. However, if PD-L1 translocates to the nucleus and leads to enhanced MRN-based DNA repair, it could also be a target used to combat chemoresistance. The reversal of chemoresistance by the downregulation of PD-L1 and NBS1 expression could lead to the use of lower concentrations of cisplatin during chemotherapy while maintaining the same or greater therapeutic profile. This should, in turn, reduce the severity of undesired side effects, such as neurotoxicity and ototoxicity.

The present study, for the first time, shows a link between PD-L1 and the MRN complex in driving chemoresistance in HNSCC. This novel finding could lead to improved patient outcomes by targeting a critical factor in treatment-related failures: chemoresistance. While this work is a step closer to developing a clinically sound therapeutic approach, further in-depth experiments should be designed to explore the nature of these relationships.

## Supplementary information

Graphical Abstract

## Data Availability

The data sets used or analysed during the current study are available from the corresponding author on request.
