# Supplementary material for: PD-L1 and MRN synergy in platinum-based chemoresistance of head and neck squamous cell carcinoma
Source: Br J Cancer. 2019 Dec 19;122(5):640–7. doi: 10.1038/s41416-019-0697-x (PMC7054324; doi:10.1038/s41416-019-0697-x)
Supplement: Supplementary file 1 — Graphical Abstract [file 41416_2019_697_MOESM1_ESM.pptx]

## Slide 1
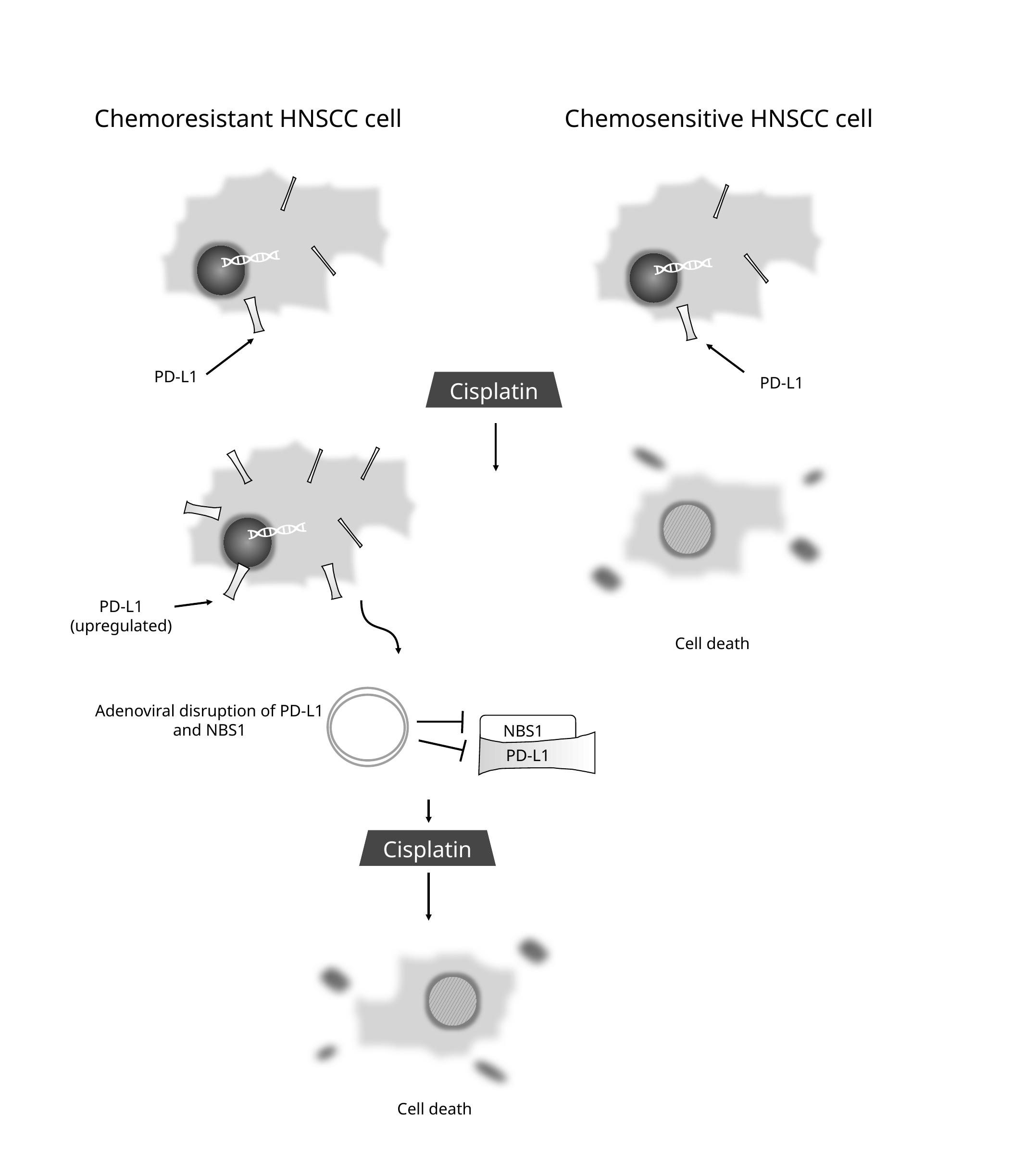

Chemosensitive HNSCC cell
Chemoresistant HNSCC cell
PD-L1
PD-L1
Cisplatin
PD-L1 (upregulated)
Cell death
Adenoviral disruption of PD-L1 and NBS1
NBS11
PD-L1
Cisplatin
Cell death
